# Machine Learning Integration of Clinical and Molecular Biomarkers to Predict Vascular Complications in Type 2 Diabetes

**DOI:** 10.3390/diagnostics16132040

**Published:** 2026-06-30

**Authors:** Gerardo García-Gil, Víctor Manuel Medina-Pérez, Joaquín Becerra-Contreras, José Alfonso Cruz-Ramos, Esteban González-Díaz, Héctor Raúl Pérez-Gómez, Kevin Javier Arellano-Arteaga, Arailym Yessenbekova, Botagoz Ussipbek, Nurzhanyat Ablaikhanova, Iryna Rusanova, Gabriela del C. López-Armas

**Affiliations:** 1 Centro de Enseñanza Técnica Industrial, Subdirección de Investigación y Extensión, Laboratorio de Biomédica/IDS, Guadalajara 44638, Mexico; ggarcia@ceti.mx; 2 Laboratorio de Sistemas Autónomos para Diseño Biotecnológico, Maestría en Ciencias en Bioingeniería y Cómputo Inteligente, Centro Universitario de Ciencias Exactas e Ingenierías, Universidad de Guadalajara, Guadalajara 44430, Mexico; victor.medina7227@alumnos.udg.mx; 3 Centro Universitario de Ciencias Biológicas y Agropecuarias, Universidad de Guadalajara, Zapopan 44600, Mexico; joaquin.becerra8216@alumnos.udg.mx; 4 Instituto de Patología Infecciosa y Experimental Francisco Ruiz Sánchez, Centro Universitario de Ciencias de la Salud, Universidad de Guadalajara, Guadalajara 44340, Mexico; jalfonso.cruz@academicos.udg.mx (J.A.C.-R.); esteban.gdiaz@academicos.udg.mx (E.G.-D.); hector.pgomez@academicos.udg.mx (H.R.P.-G.); 5 Servicio de Medicina Interna, Nuevo Hospital Civil de Guadalajara Dr. Juan I. Menchaca, Departamento de Clínicas Médicas, Centro Universitario de Ciencias de la Salud, Universidad de Guadalajara, Guadalajara 44340, Mexico; kevin.arellano@academicos.udg.mx; 6 Department of Biophysics, Biomedicine and Neuroscience, Farabi University, Farabi Av. 71, Almaty 050040, Kazakhstan; arailym.yesenbekova@kaznu.kz (A.Y.); ussipbek.botagoz@kaznu.kz (B.U.); nurzhanat.ablaihanova@kaznu.kz (N.A.); 7 Departamento de Bioquímica y Biología Molecular I, Facultad de Ciencias, Universidad de Granada, 18016 Granada, Spain; 8 Centro de Investigación Biomédica en Red de Fragilidad y Envejecimiento Saludable (CIBERFES), Instituto de Salud Carlos III (ISCIII), 28029 Madrid, Spain; 9 Centro de Investigación Biomédica, Parque Tecnológico de Ciencias de la Salud, Universidad de Granada, 18016 Granada, Spain; 10 Instituto de Investigación Biosanitaria (IBS Granada), Hospital Universitario San Cecilio, 18016 Granada, Spain; 11 Dirección del Laboratorio Estatal de Salud Pública, Secretaría de Salud Jalisco, Zapopan 45170, Mexico

**Keywords:** clinical decision support, diabetes mellitus Type 2, microvascular, macrovascular, machine learning, predictive modeling, Random Forest

## Abstract

**Background/Objectives:** Type 2 diabetes mellitus (T2DM) is a major global health challenge due to its high prevalence and association with chronic complications, highlighting the need for reliable predictive tools to support clinical decision-making. **Methods:** This study proposes a two-stage hierarchical prediction system based on a Random Forest (RF) classifier. In Stage 1, the model performs multiclass classification into healthy (H), T2DM without complications (D), and T2DM with complications (C). In Stage 2, patients classified as C are further stratified into microvascular or macrovascular complications. The dataset included 31 biochemical, molecular, inflammatory, and oxidative stress variables from Mexican and Spanish cohorts. Feature selection was performed using Pearson correlation, and feature relevance was further assessed using RF importance measures. Model training used stratified cross-validation, with additional evaluation on a hold-out set to approximate real-world performance. **Results:** The optimized RF achieved an accuracy of 92% and a macro F1-score of 0.92, outperforming baseline models, with an AUC-ROC of 0.89 for complication prediction. Key predictive features included IL-18, miR-126, duration of T2DM, HbA1c, and IL-10. **Conclusions:** The novelty of this study lies in integrating heterogeneous biomarkers within a hierarchical predictive framework, rather than in the machine learning algorithm itself. This multimodal approach, combined with interpretable machine learning techniques, is designed to deliver clinically meaningful insights for patient stratification and personalized management in T2DM.

## 1. Introduction

Type 2 diabetes mellitus (T2DM) represents a growing challenge for healthcare systems worldwide due to its high prevalence and the progressive onset of chronic complications affecting multiple organs and systems. Endothelial dysfunction resulting from chronically elevated glucose levels is a significant contributor to T2DM-related complications [1,2]. Macrovascular complications are the major problems associated with death and disabilities, with a median prevalence related to coronary artery disease (10%), peripheral arterial disease (6%), and stroke (2%). Conversely, T2DM patients also have an increased risk for microvascular complications, including neuropathy (16%) and nephropathy (15%), which together account for the highest expenditure in health systems and at the same time impair patients’ quality of life and work capacity [3]. Several factors contribute to the mechanisms underlying microvascular and macrovascular complications of T2DM, including molecular alterations, oxidative stress, genetic factors, and inflammatory processes [4,5,6]. Accurate stratification of patients by disease stage, as well as early identification of micro- and macrovascular complications, is essential for implementing personalized, effective clinical interventions in T2DM [7]. Novel drugs such as sodium-glucose cotransporter type 2 (SGLT2) inhibitors and glucagon-like peptide-1 receptor agonists (GLP-1 RAs) have demonstrated strong evidence of cardiovascular and renal protective effects [8]. Consequently, timely diagnosis and treatment strategies may reduce vascular injury and prevent vascular complications in T2DM.

Despite these advances, ML tools remain only partially explored for the comprehensive classification of T2DM micro- and macrovascular complications. Therefore, this study proposes a two-stage predictive approach based on the Random Forest (RF) algorithm to classify patients as healthy (H), T2DM without complications (D), or T2DM with complications (C), and subsequently to determine the type of complication: microvascular or macrovascular. In the first stage, the model performs a global classification of patient status; in the second, it focuses exclusively on patients with complications to improve the diagnosis. This hierarchical model enables progressive stratification that reflects the natural clinical evolution of T2DM, improving interpretability and clinical relevance compared with traditional single-stage approaches.

The proposed model integrates clinical, metabolic, inflammatory, oxidative stress, and molecular biomarkers. These heterogeneous variables were evaluated to identify the most relevant predictors associated with vascular complications in T2DM. Candidate predictors were initially filtered using Pearson correlation analysis and subsequently evaluated using RF importance measures to identify the most relevant features for classification. Notably, this study leverages a unique dataset that integrates anthropometric, clinical, inflammatory, molecular, and oxidative stress biomarkers from two international cohorts, enabling a comprehensive assessment of factors associated with vascular complications in T2DM.

Contributions of this work:

1. A two-stage machine learning framework for stratifying patients with T2DM into healthy, diabetic, and complicated categories.
2. Integration of clinical, inflammatory, molecular, and oxidative-stress biomarkers for improved prediction of microvascular and macrovascular complications.
3. Development and optimization of an RF-based predictive model with systematic feature selection and hyperparameter tuning.
4. Shapley (SHAP)-based interpretability provides meaningful clinical explanations of model predictions.

## 2. Related Work

Recent advances in machine learning (ML) have demonstrated considerable potential in medicine, providing powerful tools for predictive modeling, diagnostic support, pharmacological management, and clinical prognosis. These approaches enable the analysis of complex clinical and biomarker datasets, facilitating the identification of patterns often overlooked by traditional statistical methods [9,10,11]. Petridis et al. identified Gradient Boosting and tree-based models as some of the most effective approaches for diabetes diagnosis and prediction, highlighting the importance of incorporating emerging biomarkers to improve risk stratification and understanding of T2DM pathophysiology. Lugner et al. [12] developed an XGBoost model using UK Biobank data and ten clinical predictors, including HbA1c, BMI, age, and blood glucose levels, achieving an AUC of 0.903 for T2DM prediction. HbA1c emerged as the strongest predictor. However, the study was limited by class imbalance, the absence of biomarkers such as C-peptide and autoimmune antibodies, and the demographic homogeneity of the cohort, which may affect generalizability [13]. To address missing data and demographic variability, Abnoosian et al. [14] proposed a hybrid framework combining three diabetes datasets and an ensemble of ML and deep-learning algorithms. Their FW-CAGIN-WCAE model achieved an AUC of 0.936 ± 0.018 and identified glucose, BMI, age, pregnancy, and blood pressure as the most relevant predictors through SHAP analysis. Although highly effective, the study focused primarily on conventional clinical variables and did not incorporate emerging molecular or oxidative stress biomarkers.

Similarly, Fanoorsh et al. [15] developed hybrid machine-learning models combining clustering techniques with Gaussian Naïve Bayes classifiers. Using a dataset of 1000 individuals, the proposed approaches achieved accuracies of 0.9743 and 0.9867, with glucose identified as the most informative predictor. Despite their high performance, the models were limited by sample size, computational complexity, and the lack of emerging biomarkers associated with diabetes progression and complications.

Several studies have compared conventional ML algorithms for diabetes prediction. Bontha et al. [16] evaluated multiple models using the PIMA dataset and reported slightly superior performance for Random Forest, although at a higher computational cost. Similarly, Alghamdi et al. [17] compared Logistic Regression, CART, RF, SVM, XGBoost, and LightGBM, reporting minimal performance differences, with XGBoost achieving the highest accuracy (89%). Hasan et al. [18] proposed a hybrid CNN-LSTM architecture (DNet) that achieved 99.79% accuracy and 99.98% AUC-ROC, demonstrating the potential of deep-learning approaches for diabetes prediction. Nevertheless, these studies relied primarily on traditional clinical variables and datasets with limited representation of novel biomarkers.

In contrast, fewer studies have focused on predicting vascular complications in T2DM. Schallmoser et al. [19] investigated ML-based prediction of microvascular and macrovascular complications using electronic health records from more than 18,000 individuals. Their results identified age, blood pressure, glucose, HbA1c, creatinine, and other metabolic markers as important predictors, with predictive performance varying according to complication type. Likewise, Mora et al. [20] analyzed longitudinal data from 610,019 individuals with diabetes and compared LR, DT, RF, and XGB models for complication prediction. RF demonstrated superior performance for hypertension, myocardial complications, and retinopathy, while SHAP analysis highlighted age as a major determinant of complication risk.

## 3. Materials and Methods

### 3.1. Dataset Description

This study employed a cross-sectional design using data collected from two independent cohorts (Spanish and Mexican). The original dataset consisted of 180 participants. During preprocessing, 11 records containing missing values were excluded, resulting in a final dataset of 169 complete cases used for model development and evaluation. The exclusion of incomplete records resulted in a complete-case dataset, thereby avoiding the need for data imputation during model development.

The final analytical cohort included 84 participants from the Spanish cohort and 85 participants from the Mexican cohort. The study process included collecting and preparing clinical data from two populations diagnosed with T2DM. Spanish participants were recruited from the outpatient clinics of three hospitals in Andalusia: The Endocrinology and Nutrition Unit at University Hospital San Cecilio in Granada and the High-Resolution Hospitals in Alcalá la Real and Alcaudete. The study protocol was approved by the Ethics Committee of the Andalusian Regional Government, which oversees the participating hospitals. The registration number is 1653-N-21. The Mexican cohort included patients from The New Civil Hospital Juan I. Menchaca, who obtained ethical approval from the Ethical Committee in Mexico under Clinical Assays (reference number: 17 CI 14 039 116 COFEPRIS). Clinical laboratory tests were performed using internationally validated techniques at each hospital, and all measurements of molecular, inflammatory, and oxidative markers were conducted at the Biomedical Research Center, Health Sciences Technology Park, University of Granada, previously published by [21,22]. Both studies were conducted in accordance with the Declaration of Helsinki and the General Health Law on Research. All participants provided written informed consent before inclusion. Participants were recruited according to predefined inclusion and exclusion criteria. Patients with T2DM were classified into three clinical categories according to the presence of chronic vascular complications:

(1) T2DM without complications;
(2) T2DM with microvascular complications; and
(3) T2DM with macrovascular complications.

The presence of a history or diagnosis of defined microvascular complications:

- Diabetic retinopathy, confirmed by ophthalmologic evaluation or prior laser photocoagulation.
- Diabetic nephropathy, defined as albuminuria ≥30 mg/g creatinine or persistent estimated glomerular filtration rate < 60 mL/min/1.73 m^2^.
- Diabetic peripheral neuropathy, clinically diagnosed by neurological examination (10 g monofilament testing, reflexes assessment, and vibratory sensitivity) or confirmed by electromyography.

Macrovascular damage was established in the presence of a history or diagnosis of:

- Atherosclerotic coronary artery disease (stable or unstable angina, previous myocardial infarction, or revascularization).
- Cerebrovascular disease (transient ischemic attack or ischemic stroke).
- Peripheral arterial disease, as recorded in the medical record.

The groups were Healthy (H)—60 samples; T2DM without complications (D)—53 samples; T2DM with complications (C)—56 samples. Model development included two steps. First, a model was trained to separate samples into H, D, or C. Those in group C went to the second step, where another model distinguished microvascular (26 cases) from macrovascular (30 cases) complications. The ‘Complications’ variable was never used as a predictor. Both steps were kept strictly separate to avoid information leaking between them.

Class imbalance was considered a potential source of bias. Sensitivity analyses with class weighting and SMOTE showed limited impact on model performance. However, the relatively small sample size may affect the statistical power and stability of the second-stage model. Results should be interpreted with caution and validated in larger cohorts. To mitigate potential bias, stratified sampling was applied during model training to preserve class proportions. Data splitting occurred before model training. All preprocessing, feature selection, and model fitting were conducted exclusively on the training data at each stage to prevent information leakage.

The study was reported in accordance with the TRIPOD (Transparent Reporting of a Multivariable Prediction Model for Individual Prognosis or Diagnosis) guidelines [23]. The methodological framework consisted of a two-stage classification strategy that incorporated feature selection, perparameter optimization, and performance evaluation with standardized metrics. Supervised machine learning techniques were employed with a strong emphasis on model interpretability to support future clinical translation.

### 3.2. Data Pre-Processing

Predictor variables were evaluated using Pearson correlation coefficients with the target classes (H, D, and C). Class labels were numerically encoded before analysis. This initial dimensionality-reduction step identified variables with the strongest linear associations to the outcome and reduced redundancy and multicollinearity among candidate biomarkers [24]. The ten variables exhibiting the highest correlations were selected as candidate predictors for model development and evaluation. Pearson correlation provides a straightforward, transparent criterion for preliminary feature selection; however, it primarily detects linear relationships and may not capture nonlinear interactions. Consequently, this step served as an exploratory filter rather than a definitive feature-selection method. The selected features were subsequently evaluated using ML models capable of capturing nonlinear patterns, particularly RF, and their contributions were further assessed through SHAP-based explainability analyses. Pearson correlation-based feature selection was performed independently for each training fold during cross-validation. No information from validation folds or the hold-out set was used in feature selection, scaling, hyperparameter optimization, or model training. Although Pearson correlation was chosen for its simplicity and transparency, future studies may consider alternative feature-selection methods, such as mutual information, recursive feature elimination (RFE), permutation importance, or embedded methods, to further assess biomarker selection robustness.

The experiments were conducted using the CSV_rev1 dataset (169 observations) available in the repository. A complete-case analysis approach was adopted to ensure data consistency and avoid potential bias associated with imputation methods, particularly given the relatively small sample size and the heterogeneity of clinical and molecular variables. The source code used in this study is publicly available at https://github.com/gggvamp/T2DM (accessed on 20 June 2026).

The missing-data mechanism was not formally evaluated using Little’s MCAR test. Consequently, no assumptions were made regarding whether the missing values were Missing Completely at Random (MCAR), Missing at Random (MAR), or Missing Not at Random (MNAR). Complete-case analysis served as the primary analytical approach, while multiple imputation was conducted as a sensitivity analysis to evaluate the robustness of the findings to missing data.

**Figure 1:**
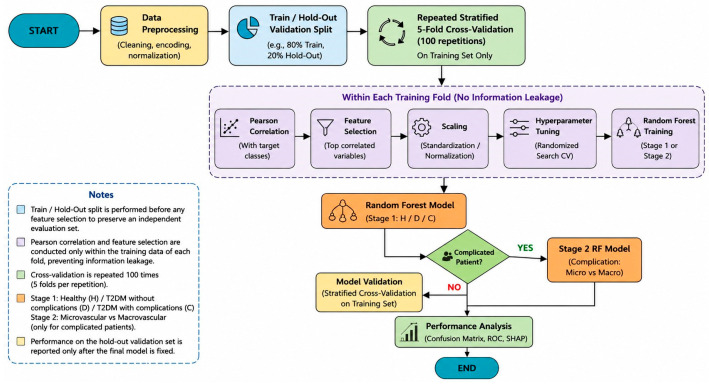
Workflow of the proposed two-stage classification system. After the train/validation split, feature selection based on Pearson correlation was conducted exclusively within the training folds during repeated stratified cross-validation, thereby preventing information leakage.

The data were divided into a training set and an internal hold-out validation set using stratified sampling to preserve proportional class representation. The dataset of 169 labeled records was split into 135 training samples (80%) and 34 samples assigned to an internal hold-out validation set (20%). Hyperparameter tuning was performed on the training set using GridSearchCV with stratified 5-fold cross-validation. Model performance was subsequently estimated using repeated stratified 5-fold cross-validation (100 repetitions) on the training set and finally assessed on the independent hold-out validation set to evaluate generalization performance on unseen data. The validation set is an internal partition of the same dataset and does not represent an external validation cohort. Future studies should incorporate dedicated multicollinearity analyses to strengthen feature selection and biomarker interpretation further. This validation strategy ensures consistency between model optimization and evaluation while minimizing the risk of data leakage, as illustrated in Figure 1.

The predictive model was constructed using an RF classifier implemented in Python with the scikit-learn library (v1.3.0) [25]. Subsequently, StandardScaler was fitted exclusively on the corresponding training data and then applied to the associated validation fold.

**Figure 2:**
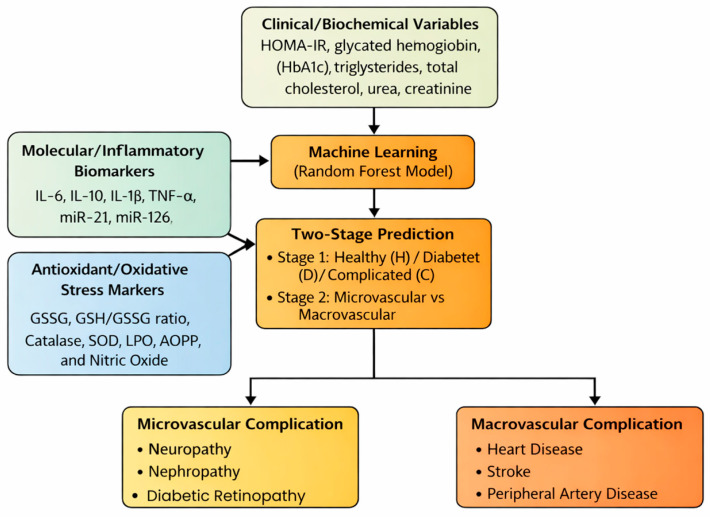
Biomarkers separated by subsets: Clinical, Molecular, Inflammatory, and Antioxidant systems.

To assess the predictive contribution of different biomarker categories, the dataset was divided into three subsets. Each subset was built upon a core set of standard variables to ensure a comparable baseline across models. This core set included anthropometric data (age, sex, body mass index [BMI]), hypertension status, family history of T2DM, physical activity, smoking status, and disease duration (years). A specific group of biomarkers was added to this common core to form each of the three subsets (see Figure 2).

1. Clinical-Biochemical subset: homeostatic model assessment of insulin resistance (HOMA-IR), glycated hemoglobin (HbA1c), triglycerides, total cholesterol, urea, and creatinine.
2. Molecular and inflammatory subset: interleukin-6 (IL-6), IL-10, IL-18, tumor necrosis factor alpha (TNF-α), miR-21-5p, and miR-126-3p.
3. Oxidative-Stress and Antioxidant subset: oxidized glutathione (GSSG), reduced glutathione (GSH), GSH/GSSG ratio, glutathione reductase, glutathione peroxidase, catalase, superoxide dismutase (SOD), glucose-6-phosphate dehydrogenase (G6PDH), lipid peroxidation (LPO), advanced oxidation protein products (AOPP), and nitric oxide (NO).

### 3.3. Machine Learning Algorithms

Each of the three data subsets was independently used to train and evaluate ten ML algorithms: RF, DT, SVM, Multilayer Perceptron (MLP), Naïve Bayes (NB), Linear Discriminant Analysis (LDA), LR, and Quadratic Discriminant Analysis (QDA). Model performance was assessed using standard classification metrics, including accuracy, precision, recall, and F1-score, enabling a direct comparison of the predictive capabilities of each variable group.

For Stage 1, the search space included n_estimators {100, 500}, max_depth {5, 10, None}, and min_samples_split {2, 5}. For Stage 2, the search space included n_estimators {100, 500}, max_depth {3, 5, None}, and min_samples_split {2, 5}. All Random Forest models used class_weight set to “balanced” and random_state set to 42. The hyperparameters yielding the highest mean Macro F1 score during cross-validation were used to train the final model for each stage [26].

Among the evaluated algorithms, RF was selected as the final predictive model because it achieved the best overall classification performance while providing robustness to nonlinear relationships, resistance to overfitting, and straightforward feature-importance estimation.

**Figure 3:**
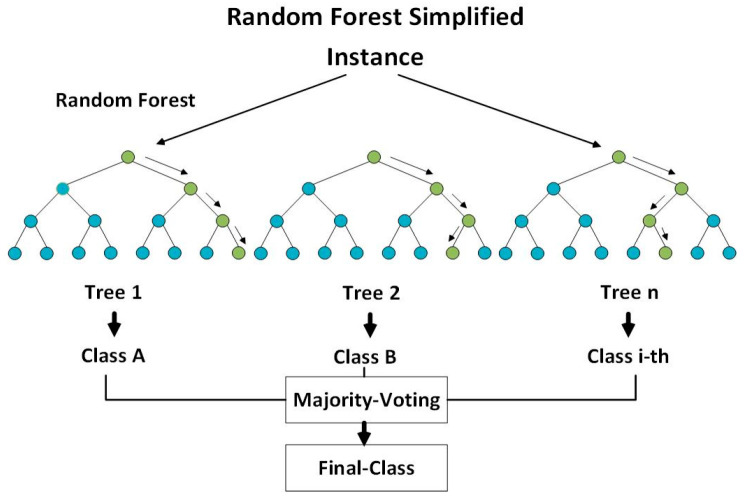
Diagram of RF simplified.

Decision Tree (DT) was included as a baseline classifier for performance comparison [27]. The RF algorithm, proposed by Breiman et al. in 2001 [28], is an ensemble-learning algorithm that combines multiple decision trees trained on bootstrap samples of the data. At each node, a random subset of predictors is considered for splitting, reducing correlations among trees and improving generalization performance. Final predictions are obtained through majority voting across trees. RF is particularly suitable for biomedical datasets because it can model nonlinear relationships, handle mixed data types, reduce overfitting, and provide measures of variable importance. Key hyperparameters include the number of trees (n_estimators), maximum tree depth (max_depth), minimum samples required for node splitting (min_samples_split), and the number of candidate variables evaluated at each split (max_features) [29,30]. A simplified schematic diagram of the RF algorithm is shown in Figure 3.

Among its main advantages are robustness to overfitting, strong predictive performance, and good generalization capability. These characteristics facilitate clinical interpretation by enabling an objective estimate of the relative importance of predictor variables, an aspect essential for evidence-based medical decision-making. Class imbalance was addressed using class weights during model training and further evaluated through sensitivity analyses with the Synthetic Minority Over-sampling Technique (SMOTE). From a clinical perspective, this approach prioritizes accurate identification of patients with complications, as failing to detect them can have significant clinical consequences. Finally, the following sections detail the key mathematical components underlying the algorithm [31,32].

### 3.4. Mathematical Foundations of Random Forest

- Classification by majority vote

Given a set of B independent decision trees, each tree generates a prediction *T_B_* (x) for observation *x*. The final prediction of the model is obtained by majority vote (mode of individual predictions):

(1) $$RF(x)\;=\;mode\{\;T_{1}(x),T_{2}(x),\ldots ,T_{B}(x)\}$$

where RF denotes the class predicted by the model; *T_B_*(x) represents the prediction of the b-th tree for the feature vector x; and B indicates the total number of trees in the ensemble.

B. Estimated probabilities by class

RF also allows us to estimate the probability of an observation x belonging to a specific class. k ∈ {0, 1, 2}, by averaging individual predictions:

(2) $$P(y\;=\;k\;|\;x)\;=\frac{1}{B}\;\sum_{b=1}^{B}I\left(T_{b}\left(x\right)=k\right)$$

where B denotes the total number of trees in the ensemble, *T_b_* (x) represents the prediction of the b-th tree for the input vector x, and I(*T_b_*(x) = k) is an indicator function that takes the value 1 if the b-th tree assigns class k, and 0 otherwise.

C. Measurement of impurity (Gini index)

During tree construction, node divisions are based on impurity criteria. One of the most common is the Gini impurity, defined as:

(3) $$G\left(t\right)=1-\sum_{i=1}^{C}p_{i}^{2}$$

where G(t) denotes the impurity at node t, C refers to the total number of classes considered in the split, and *p_i_* indicates the proportion of samples assigned to class i at that node. This index reaches its minimum value (zero) when the node is pure, that is, when all observations belong to the same class.

D. Gain on impurity (division criterion)

To select the best split at a node, the impurity reduction (gain) from splitting a parent node into two child nodes is evaluated, *T_L_* and *T_R_*:

(4) $$\Delta_{i\left(t\right)}=i\left(t\right)-\left[\frac{N_{L}}{N}i\left(T_{L}\right)+\frac{N_{R}}{N}i\left(T_{R}\right)\right]$$

where i(t) denotes the impurity of the parent node; i(*T_L_*) and i(*T_R_*) correspond to the impurities of the left and right child nodes; *N_L_* and *N_R_* represent the number of observations assigned to the child nodes; and N is the total number of observations in the parent node.

E. Importance of predictor variables

The importance of a variable *X_j_* is calculated as the weighted sum of the impurity reductions generated by that variable in all nodes and trees in which it participates. This metric, known as Mean Decrease Impurity (MDI), is defined as:

(5) $$Imp(X_{j})=\frac{1}{B}\;\sum_{b=1}^{B}\sum_{t\in \;T_{b};v\left(t\right)=X_{j}}p(t)\cdot \Delta_{i\left(t\right)}$$

where v(t) = *X_j_* indicates that the variable *X_j_* is used to split node t; p(t) represents the proportion of samples that reach node t; and Δ*_i_*_(_*_t_*_)_ denotes the impurity reduction achieved by the split at that node.

This measure allows us to quantify each variable’s relevance to the model, which is essential in the biomedical context, where interpreting the contributions of different clinical biomarkers is necessary.

### 3.5. Metrics for Model Performance

#### 3.5.1. Confusion Matrix

Classification model performance was evaluated using metrics derived from the confusion matrix in Table 1, which allows the performance of a classifier to be described based on four categories:

The primary metric used was F1 macro, which is appropriate for class-imbalance scenarios. In addition, accuracy, precision, and recall were reported, and to assess stability across folds, the standard deviation of F1 (Std. F1) was computed.

#### 3.5.2. Random Forest Model Explanation with SHAP

To improve transparency and interpretability, we complemented the model’s performance metrics with SHAP analyses. SHAP is a game-theory–based framework that quantifies each -*-/*- individual contribution to the model output. This approach allows for the decomposition of each prediction into additive components, indicating whether a given variable increases or decreases the probability of complications [33,34].

Formally, the prediction of a model *f*(*x*) for an instance *x* can be expressed as:

(6) $$F\left(x\right)=E[f\left(x\right)]+\sum_{i=1}^{M}\emptyset_{i}$$

where *E*[*f*(*x*)] is the expected prediction, and $\phi_{i}$ represents the contribution of feature *i*. The Shapley value for feature *i* is defined as:

(7) $$\emptyset_{i}\left(f,x\right)=\sum_{S\subseteq F\{i\}}\frac{\left|S\right|!\left(M-\left|S\right|-1\right)!}{M!}[E(f(x)\left|x_{S⋃\{i\}})-E(f(x)\right|x_{s})]$$

where *F* denotes the set of all features, *S* is a subset of features not containing feature *i*, and *E*(*f*(*x*) | *x_s_*) represents the expected prediction conditioned on the subset *S*. This formulation ensures that the contribution of each feature is fairly distributed across all possible feature coalitions.

SHAP values satisfy key theoretical properties, including local accuracy, consistency, and missingness, ensuring reliable attribution of feature contributions. SHAP analysis provided both global and local interpretability, identifying the most influential predictors and revealing how specific variables affected individual predictions. The resulting feature-importance patterns were consistent with established knowledge of T2DM progression, particularly regarding disease duration, inflammatory biomarkers, glycemic control, and oxidative stress.

## 4. Results

This section presents the performance of the ML models, including feature-selection results, classification performance, and model interpretability analyses. Model evaluation was conducted using correlation matrices, confusion matrices, AUC-ROC metrics, and SHAP explanations. For the multiclass classification task, ROC analysis was performed using a One-vs-Rest (OvR) strategy, and the reported macro-average AUC summarizes the overall discrimination performance across the three classes.

### 4.1. Pearson Correlation Matrix

**Figure 4:**
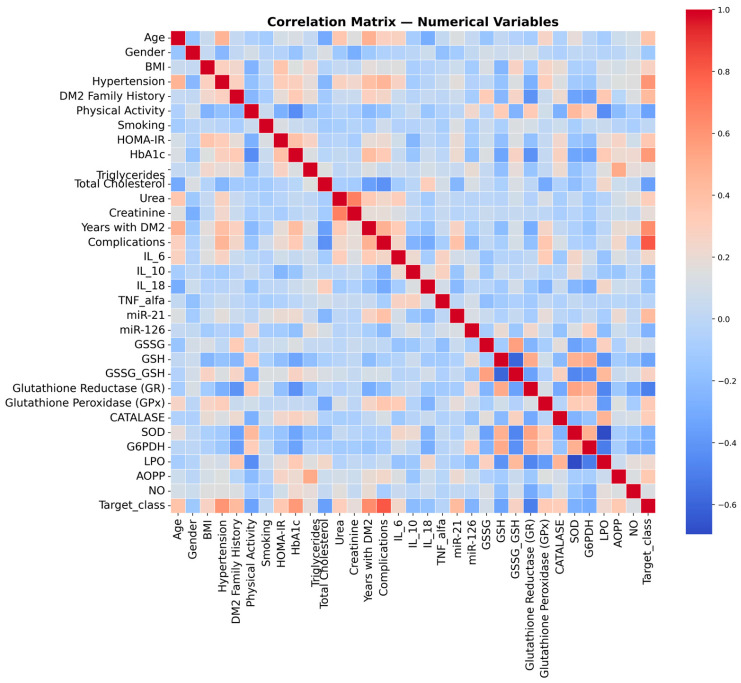
Pearson’s correlation matrix of numerical variables in the study.

Pearson correlation analysis was performed as an initial exploratory step to evaluate the linear association between the encoded class variable and the candidate predictors. Figure 4 presents the correlation matrix of the analyzed variables, highlighting the features that showed the strongest associations with the three classification categories: H, D, and C. These results supported the preliminary identification of potentially relevant predictors for subsequent model development.

This analysis highlights the variables that show the strongest linear associations with the encoded class variable and provides an initial indication of their potential relevance to patient stratification. The corresponding absolute correlation coefficients are presented in Table 2.

#### 4.1.1. Interpretation of Pearson Correlation Results

(1) Variables with high positive correlation

Among the variables with the strongest positive association are “Hypertension,” “HbA1c,” “Years with T2DM,” “Family history of T2DM (YES/NO),” and “Age.” This indicates that the presence of hypertension, higher glycosylated hemoglobin values, longer duration of disease, family history, and older age are strongly related to disease progression and the development of complications.

(2) Relevant metabolic and inflammatory variables

HOMA-IR, body mass index (BMI), and miR-21 also showed significant associations, highlighting the roles of insulin resistance, excess body weight, and molecular biomarkers in the clinical stratification of patients.

(3) Variables with significant negative correlation

Markers such as “total cholesterol,” “IL-18,” and “glutathione reductase” show notable negative correlations, suggesting that lower values of these markers may be associated with advanced or more complicated stages of the disease. However, their biological significance should be interpreted with caution and in the context of clinical findings.

#### 4.1.2. Top Class Correlated Variables (H, D, C)

**Figure 5:**
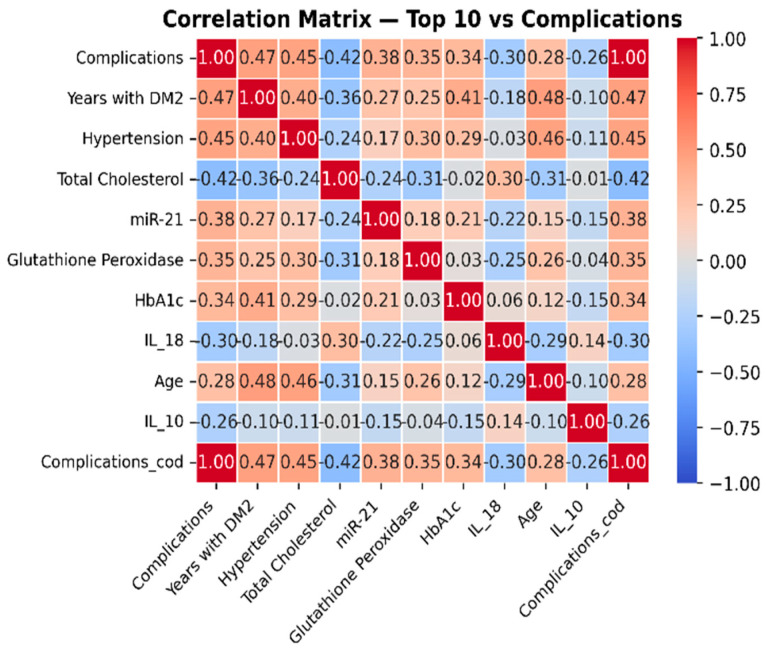
Correlation matrix of key predictors for complications in T2DM.

Figure 5 presents the correlation structure between the candidate predictors and the outcome variable. Variables directly defining the outcome (Complications and Complications_cod) are displayed only as reference variables in the correlation analysis and were excluded from all feature-selection, training, and validation procedures to avoid information leakage.

Correlation analysis identified the clinical and biochemical factors most closely associated with complications in patients with T2DM. Table 3 presents the correlation matrix results.

The complications variable showed a perfect correlation coefficient (r = 1.0) with itself, since any variable has an identical correlation with itself. Among the biological biomarkers, the duration of T2DM in years showed the highest correlation (r = 0.474). On the other hand, hypertension showed a significant correlation (r = 0.454), reinforcing its recognized role as a cardiovascular risk factor and predictor of macrovascular complications. Similarly, the total cholesterol (r = 0.421) emerged as an essential clinical determinant, consistent with its well-established role in vascular damage in T2DM.

Novel molecular biomarkers associated with T2DM have also emerged. miR-21 (r = 0.382), a microRNA involved in inflammation, fibrosis, and vascular damage, demonstrated potential as an emerging predictor of complications in T2DM. Likewise, oxidative stress markers were identified as relevant predictors: glutathione peroxidase (r = 0.354), an antioxidant enzyme, showed a significant correlation, highlighting the role of oxidative stress in vascular damage.

Consistent with its established role as a marker of long-term glycemic control, HbA1c (r = 0.342536) reaffirmed its clinical relevance, with higher levels associated with an increased risk of complications. Interestingly, proinflammatory cytokine IL-18 (r = 0.302) and anti-inflammatory cytokine IL-10 (r = 0.265) may reflect compensatory mechanisms against chronic inflammation, a common feature of complicated T2DM. Finally, age also demonstrated a moderate association with complication risk (r = 0.284).

Taken together, these findings suggest that both traditional clinical variables (years with T2DM, hypertension, cholesterol) and the most recent molecular and biochemical biomarkers (miR-21, glutathione peroxidase, IL-18, IL-10) contribute significantly to stratifying patients. The variables showing strong correlations align with key pathological mechanisms previously recognized in the literature, supporting their inclusion in predictive models of complication risk [35].

### 4.2. Statistical Analysis

To evaluate the robustness of model performance, a non-parametric bootstrap resampling procedure with 1000 iterations was applied to the internal hold-out validation set. For each iteration b = 1,…,B, a bootstrap sample D^(b)^ of the same size as the internal validation set (*n* = 34) was generated by sampling with replacement from the validation data.

The 95% confidence intervals (95% CI) were estimated using the percentile method, where ${\hat{\theta }}_{2.5}$ and ${\hat{\theta }}_{97.5}$ correspond to the 2.5th and 97.5th percentiles of the empirical distribution of ${\hat{\theta }}^{\left(b\right)}$. This non-parametric approach allows estimation of model variability without assuming a specific underlying distribution and is particularly suitable for datasets of limited size.

**Figure 6:**
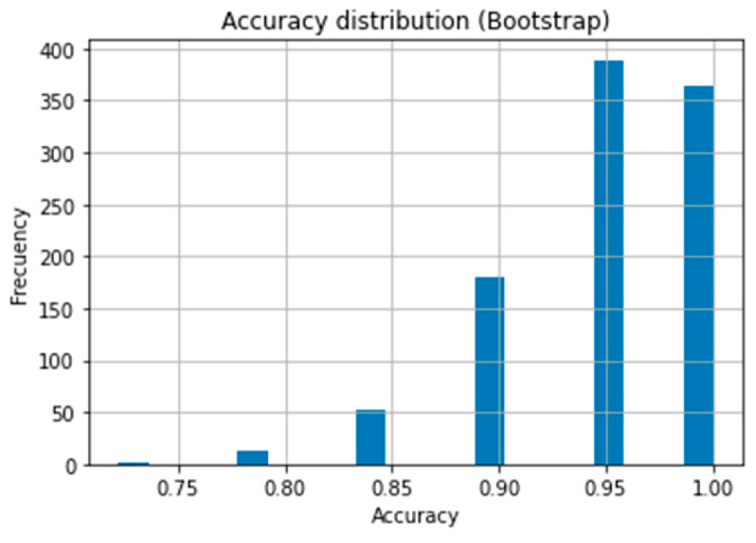
Distribution of model accuracy obtained through bootstrap resampling.

The resulting bootstrap distribution is shown in Figure 6. A clear concentration of values around high accuracy levels can be observed, indicating stable model performance and low variability across bootstrap estimates.

Additionally, the AUC–ROC was calculated in Python using the predicted class probabilities. ROC curves graphically represent the trade-off between sensitivity and specificity across decision thresholds. The AUC summarizes a classifier’s overall ability to distinguish between classes, with higher values indicating better performance. The resulting AUC–ROC curves were generated for the best- and worst-performing RF models.

To explore potential differences among classifiers, the distributions of accuracy scores obtained across cross-validation folds were compared using the Wilcoxon signed-rank test (Table 4). These statistical comparisons should be regarded as exploratory and interpreted with caution. Consequently, the results are not intended to establish definitive statistical superiority among classifiers but rather to provide an additional assessment of performance consistency.

To evaluate the robustness of the selected model, a non-parametric bootstrap analysis was performed. The resulting accuracy distribution was concentrated around high values and exhibited relatively low variability, indicating stable predictive performance. Although a small proportion of bootstrap samples yielded lower accuracy, these instances were infrequent and did not substantially affect the overall performance estimates. The corresponding 95% bootstrap confidence interval indicated stable predictive performance across resampled datasets, supporting the reliability of the proposed model within the study population.

As a complementary evaluation, ROC analysis was performed. The RF classifier achieved a micro-averaged AUC of 0.95, indicating excellent discriminative capability across the evaluated classes. Nevertheless, this result should be interpreted with caution, given the relatively small sample size and the lack of external validation.

**Figure 7:**
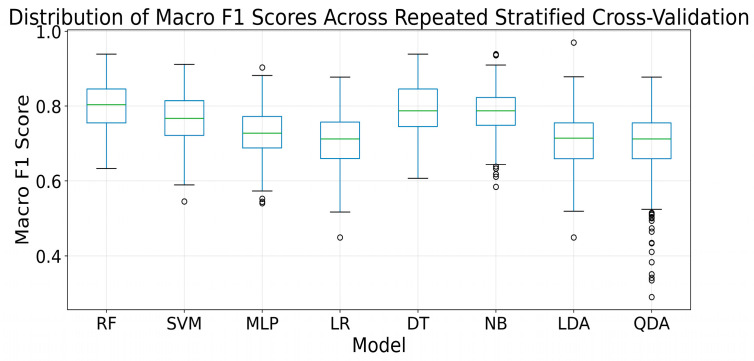
Distribution of Macro F1 scores across repeated stratified cross-validation experiments (5 folds × 100 repetitions). Boxplots represent the median, interquartile range, and dispersion of model performance. The figure illustrates the variability and stability of the evaluated classifiers and complements the statistical comparisons presented in Table 5.

In contrast, no statistically significant differences were observed between ML’s. Given the limited number of cross-validation folds and the relatively small sample size, these findings should be interpreted as exploratory rather than conclusive evidence of classifier superiority. The distribution of model performance across the different cross-validation folds is presented in Figure 7.

These statistical comparisons should be interpreted with caution. The Wilcoxon signed-rank test was performed on performance scores obtained from the same stratified 5-fold cross-validation procedure used throughout the study, yielding only five observations per model for hypothesis testing. While this approach preserves methodological consistency with the reported classification results, the limited number of folds may reduce the test’s stability and statistical power. Consequently, the Wilcoxon analysis should be regarded as an exploratory assessment of performance consistency rather than definitive evidence of classifier superiority. The primary conclusions of this study are based on the predictive performance metrics reported in Table 5.

### 4.3. Machine Learning Models’ Performance

Several ML classifiers were trained and compared using the selected features. A repeated stratified cross-validation was implemented to ensure that each training and validation subset preserved the original class proportions (H, D, and C). Cross-validation provides a reliable estimate of model performance on unseen data, reduces the risk of overfitting, and enables a consistent comparison among competing classifiers.

Table 5 compares the performance of the evaluated ML models for diagnostic classification of patients (H, D, and C). The metrics used were Macro F1-score and its standard deviation, offering a balanced view of predictive performance across the three classes. Macro F1-score was chosen for its ability to reflect both precision and recall while minimizing the impact of class imbalance. RF achieved the highest Macro F1-score, with DT and NB performing slightly lower. These results indicate that tree-based models delivered competitive and consistent performance across repeated validation, while other classifiers showed lower average scores and greater variability.

### 4.4. Calibration Analysis

**Figure 8:**
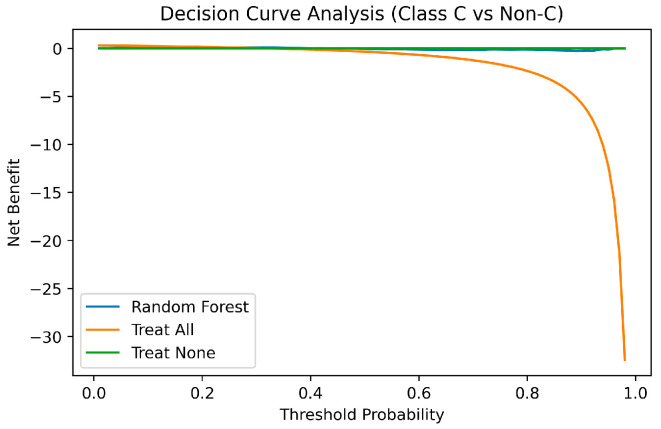
Decision Curve Analysis (DCA) of the Random Forest model for identifying patients with diabetic complications (Class C).

To assess the reliability of predicted probabilities, a calibration analysis was performed to identify patients with diabetic complications (Class C). The RF model obtained a calibration intercept of 0.003 and a calibration slope of 0.961, indicating excellent agreement between predicted and observed risks, with minimal evidence of systematic bias or overadjustment. Additionally, the Brier score was 0.152, indicating good probabilistic accuracy of the model predictions. The clinical utility of the model was further evaluated using Decision Curve Analysis (DCA), as shown in Figure 8.

The DCA demonstrated that the RF model achieved a non-negative net benefit across most clinically relevant threshold probabilities. Furthermore, the model outperformed the “treat-all” strategy across a wide range of thresholds, suggesting potential clinical utility in identifying patients with diabetic complications while avoiding unnecessary interventions.

The primary comparison metric was the macro F1-score, which is particularly appropriate for multiclass classification problems because it assigns equal weight to precision and recall across all diagnostic categories. The results show that RF (F1 = 0.9419) achieved the highest predictive performance among the evaluated classifiers, followed by DT (F1 = 0.9066), SVM (F1 = 0.8711), MP (F1 = 0.8462), and NB (F1 = 0.8318), which demonstrated moderate predictive performance.

Linear models, including LDA (F1 = 0.8046) and LR (F1 = 0.7951), achieved comparatively lower results. QDA exhibited the weakest performance (F1 = 0.4663), indicating limited suitability for this classification task.

Overall, the RF model achieved the highest performance among the evaluated classifiers, consistently outperforming the others across accuracy, precision, recall, and F1-score. In addition to its predictive performance, the model provides interpretable estimates of feature importance, supporting its potential utility as a clinical decision-support tool for the stratification and management of patients with T2DM.

### 4.5. The Clinical and Scientific Value of Random Forest for Personalized Medicine in Type 2 Diabetes Mellitus

The RF model was implemented to classify patients with T2DM into three clinical categories: H, D, and C.

The methodological flow comprised:

- Selection of clinical, biochemical, and molecular variables through correlation analysis.
- Training and validation of the model using stratified cross-validation.
- Systematic comparison of performance against classic supervised classification algorithms.

**Figure 9:**
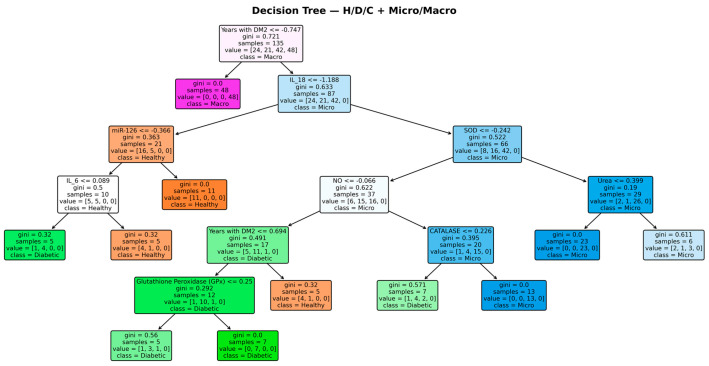
The tree shown is representative of the entire forest. It is not the final model, but it allows us to observe how the system learns clinical patterns. Each node contains the variable used for division, the cutoff point, the Gini index, the number of patients, and the predominant class. The final classes in this tree are: Healthy, Diabetic, Complicated-Micro, and Complicated-Macro.

The results indicate that the RF algorithm achieved high predictive performance in multiclass prediction and identified key predictors of complication development, thereby suggesting potential clinical and research value for personalized medicine in T2DM. A vector of clinical, biochemical, and molecular characteristics represented each patient. For each diagnostic class k ∈ {0 (H), 1 (D), 2 (C)}, the model estimated the probability of group membership using the RF algorithm’s internal voting mechanism. When the predicted class was “Complicated Diabetic (C),” a secondary classification model, specifically trained to predict the complication subtype (microvascular or macrovascular), was applied (See Figure 9). This system provides both categorical predictions and risk probabilities for each class, thereby enhancing clinical interpretation based on each patient’s probabilistic profile. In this approach, the assigned class corresponds to the category with the highest estimated probability, calculated as the proportion of trees in the forest that predict each category, or, equivalently, the category with the most votes among all base classifiers.

This procedure enables robust classification and provides a probabilistic interpretation of diagnostic certainty, which is valuable for informed clinical decision-making. The hierarchical prediction architecture not only detects complications but also predicts their specific nature, offering an advanced tool for precision medicine and individualized clinical follow-up. After identifying the clinical variables standardized across the study, the differential behavior of molecular biomarkers, such as inflammatory cytokines and microRNAs, and antioxidant systems was analyzed in relation to metabolic status and the presence of complications. This analysis helps elucidate the pathophysiological mechanisms of T2DM and its vascular complications beyond traditional risk factors.

The DT begins with “years since T2DM diagnosis,” which the model identifies as the primary splitting variable. Patients with shorter disease duration tend to remain in the non-complicated category, whereas longer disease duration is associated with an increased risk of complications. This coincides with the disease’s pathophysiology: metabolic and vascular damage accumulates progressively over time. Along the right branch, corresponding to a longer duration of diabetes, the model evaluates IL-18, an inflammatory marker. Elevated IL-18 levels are associated with a higher risk of complications, particularly microvascular complications. Subsequently, variables such as SOD, catalase, NO, and urea serve as additional decision nodes, reflecting processes associated with oxidative stress and renal impairment. These features may indicate progression toward vascular damage and microangiopathy [36]. In contrast, the left branch, corresponding to a shorter disease evolution time, includes miR-126, IL-6, TNF-α, and HbA1c as early discriminative markers. When these markers remain low, the model classifies the patient as healthy. When they begin to rise, even though there is still no clear evidence of structural damage, the model classifies the patient as diabetic.

Overall, the DT shows a clinically coherent progression, in which patients transition from healthy status to diabetes and subsequently to microvascular and macrovascular complications as disease duration, inflammation, oxidative stress, and metabolic deterioration increase.

### 4.6. Interpretation of the Illustrative Decision Tree

In the individual tree extracted from the RF model, all variables have been standardized (mean = 0, standard deviation = 1), allowing a homogeneous comparison of cut-off points across variables initially expressed in different clinical units.

(1) Level 1—Years with diabetes (Years with T2DM)

The first division of the tree is based on the progression of T2DM. Patients with less time with the disease tend to remain in:

• Healthy      • Diabetic with complications

Meanwhile, patients with more time with T2DM are more likely to progress to:

• Complicated-Micro      • Complicated-Macro

F rom a clinical point of view, this is consistent: the longer the duration of T2DM, the greater the accumulation of metabolic, vascular, and renal damage.

Nodes with a Gini close to 0 indicate subgroups with almost perfect separation, i.e., the duration of evolution alone effectively distinguishes specific profiles.

(2) Level 2—IL-18 (systemic inflammation)

In branches where the disease is already prolonged, the model evaluates IL-18, an inflammatory biomarker. The model typically classifies low or moderate IL-18 values as T2DM. High IL-18 values clearly increase the likelihood of Complications, especially microvascular ones. This supports a well-known concept: persistent inflammation contributes to endothelial damage and the progression toward complications.

(3) Level 3—oxidative stress (SOD, catalase, NO, and LPO)

At deeper levels of the tree, markers of oxidative stress appear: SOD, Catalase, NO, and LPO. These help to differentiate:

1. T2DM without complications;
2. Complicated-Micro;
3. Complicated-Macro.

The pattern observed is:

Greater oxidative stress, greater likelihood of complications, and especially:

1. microvascular when altered antioxidant defenses predominate;
2. macrovascular when inflammation + lipid damage are combined.

This is physiologically consistent: chronic hyperglycemia and inflammation deplete antioxidant defenses and facilitate vascular injury.

(4) Role of HbA1c, Age, and Hypertension

In parallel branches, the tree uses:

HbA1c: discriminates between adequate metabolic control and poor control.

Age: reflects cumulative burden and vascular fragility.

Hypertension acts as a cofactor, increasing risk when inflammation and oxidative stress are already present. They are not isolating determinants, but their combination significantly increases risk.

(5) Terminal nodes and gini = 0

Leaves with Gini = 0 indicate:

All patients in the node belong to the same class.

In these subgroups, specific combinations such as:

- many years with T2DM;
- elevated IL-18;
- altered oxidative markers;

almost certainly identify complicated patients.

Where:

- Each node represents a condition based on the value of a variable.
- Each branch indicates a possible response to that condition.
- The terminal leaves contain the predicted class, the distribution of observations, and the residual impurity of the node.

### 4.7. Variable Importance in the Random Forest Model

The relative importance of each predictive variable was evaluated using the mean decrease in impurity (MDI) in the RF ensemble. The most influential variables in the multiclass classification are shown in Table 6 with the first stage of RF (H, D, and C).

### 4.8. Confusion Matrix—General Classification

**Figure 10:**
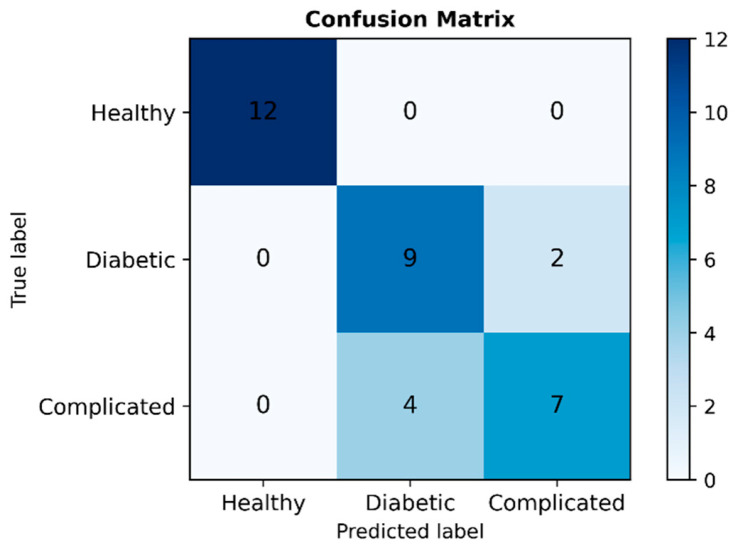
Confusion matrix for the RF.

The confusion matrix in Figure 10 summarizes the classification results in the test set (20%). The model showed robust performance in distinguishing between the three classes, with higher accuracy for healthy individuals and uncomplicated diabetics. Misclassification was more frequent among people with T2DM and those with complicated diabetes, consistent with the clinical overlap between these populations.

The model showed adequate overall performance (accuracy = 0.92). Sensitivity was excellent for class H (recall = 1.00), high for D (0.82), and moderate for C (0.64). Precision was higher in S (1.00) and C (0.78), but lower in D (0.69). Overall, these results indicate that the model effectively discriminates among the three clinical states. However, some underdetection was observed in the complications class, which is clinically relevant and suggests that threshold adjustment may be necessary to prioritize sensitivity for this category. Detailed results are presented in Table 7.

Given the clinical importance of the “Complicated (C)” class, the probabilistic decision threshold was further analyzed. The default classification threshold of P(C) ≥ 0.50 was used for model evaluation; however, this criterion led to underdetection of patients with complications. To explore potential improvements in sensitivity, a lower threshold (0.45) was evaluated as an additional analysis. This adjustment was not used during model training or selection and should be interpreted as exploratory. This modification increased sensitivity for class C from 0.64 to approximately 0.82, enabling identification of more at-risk patients, albeit with a moderate rise in false positives, primarily from class D. Clinically, this approach is preferable because failing to identify a complicated patient poses a greater risk than subjecting a patient incorrectly classified as complicated to additional monitoring. Thus, adjusting the threshold enhances the system’s practical value as a decision-support tool in medical settings.

The classifier distinguishes Healthy individuals from other classes with near-perfect accuracy and maintains strong performance in differentiating Diabetic from Complicated cases, with only minor residual errors (2↔4 cases). If the primary clinical objective is to avoid missing complicated patients, the decision threshold for the Complicated class can be lowered, or the cost of false negatives increased. Conversely, if reducing the overdiagnosis of complications is prioritized, the threshold should be raised.

### 4.9. Classification of Microvascular vs. Macrovascular Complications

The second stage of the proposed hierarchical framework focuses on classifying vascular complications into microvascular and macrovascular categories. This analysis was performed exclusively on patients previously classified as “C” in Stage 1. The Stage 2 dataset included 56 patients, of whom 26 had microvascular complications, and 30 had macrovascular complications, indicating a notable class imbalance, as can be seen in Table 8. Additionally, the prominent variables in the multiclass classification are shown in Table 9 for the second stage of RF (micro and macrovascular classification).

The model achieved an overall accuracy of 0.80. It demonstrated strong performance in identifying macrovascular complications, with a recall of 1.00 and an F1-score of 0.88, indicating that all macrovascular cases were correctly classified. These results are presented in Table 10.

In contrast, performance on microvascular complications was limited, with a recall of 0.14 and an F1 Score of 0.25. The confusion matrix (Table 9) revealed that six out of seven microvascular cases were misclassified as macrovascular. This performance asymmetry is primarily due to the reduced number of microvascular cases, which limits the model’s ability to learn representative patterns for this class. Despite the use of class balancing strategies, the imbalance remains a significant challenge. These results suggest that while the proposed framework is effective in identifying macrovascular complications, further improvements and larger, more balanced datasets are required to enhance the detection of microvascular conditions.

### 4.10. ROC Curve for Class

**Figure 11:**
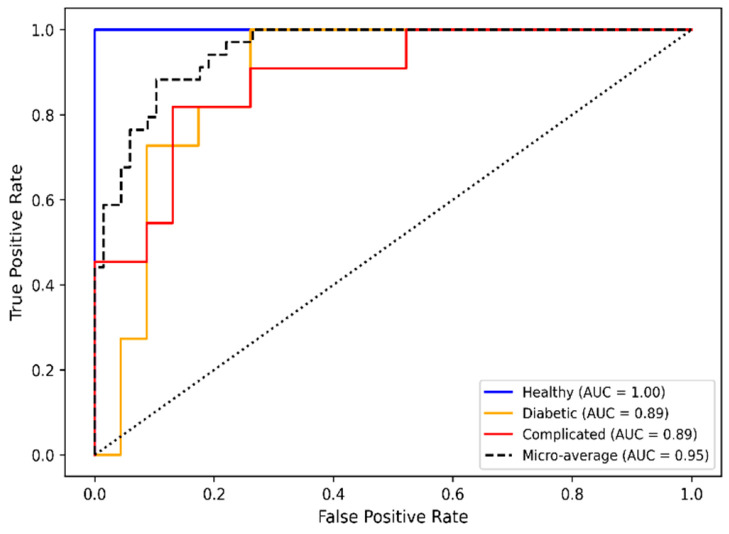
ROC curves for the RF performance in Healthy, AUC = 1.00, Diabetic, AUC = 0.89, and T2DM complications, AUC = 0.89. Each model was constructed using the features of each group, as defined in the dataset description section.

Figure 11 presents the ROC curves for each class. For the Healthy class, the AUC reached 1.00, indicating virtually perfect performance. The model consistently distinguishes healthy subjects from disease states, as also reflected in the confusion matrix, suggesting minimal misclassification of healthy individuals.

For the Diabetic class, the AUC of 0.89 indicates strong discriminative power. The model correctly identifies most diabetic patients without complications, although some overlap with the complication class is observed, explaining instances reclassified as “Complicated.” This behavior may be interpreted as a conservative classification tendency that prioritizes clinical safety.

Similarly, the curve for the Complicated class achieved an AUC of 0.89, suggesting the model is effective at detecting patients with significant complications. However, an AUC below 1.0 suggests that some patients with complications may still be misclassified as diabetic without complications, consistent with the confusion matrix.

Finally, the micro-average AUC of 0.95 summarizes the overall model’s performance in multi-class classification. This high value indicates strong discriminative power in distinguishing among the three clinical stages of diabetes progression, reinforcing the model’s potential utility as a clinical decision-support tool.

Although Precision–Recall analysis was explored during model development, ROC-AUC, class-wise precision, recall, and F1-score were retained as the primary evaluation metrics because they provide a more comprehensive assessment of multiclass performance in the present dataset.

### 4.11. Shapley Additive Explanations in ML Algorithm

(1) SHAP diagnosis of healthy patients

The SHAP graphs for the “Healthy” class help us understand why the model classifies a patient as healthy rather than diabetic or complicated. Instead of simply issuing a prediction, the model shows the individual contribution of each variable to the result. In this context, negative SHAP values shift the prediction toward the healthy category, while positive values reduce the probability of remaining in that class. This makes the model an explanatory tool, useful for both clinicians and researchers. It is important to note that SHAP values provide insights into the model’s decision-making process but do not imply causal relationships. Therefore, the identified feature contributions should be interpreted as associations learned by the model rather than direct evidence of underlying biological mechanisms.

**Figure 12:**
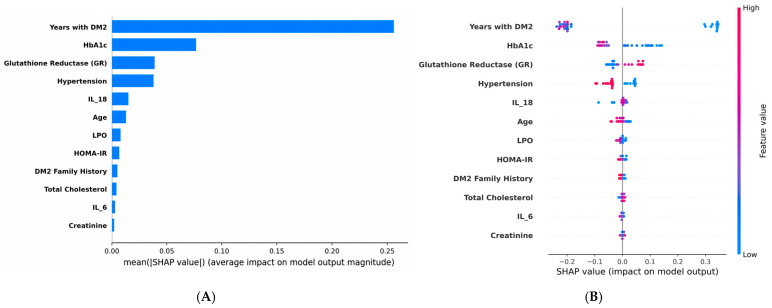
(**A**) Global features are important in the healthy class (SHAP). (**B**) Feature impact on predicting the healthy class (SHAP).

Figure 12A summarizes the overall importance of the variables and shows that the duration of diabetes (Years with T2DM) is the main determinant. Most patients classified as healthy have few or no years with a diagnosis, which is fully consistent with the natural course of the disease. The second most relevant factor is HbA1c; low values indicate adequate glycemic control and favor remaining in the healthy category, while higher values shift the patient toward pathological states. Additionally, more conserved glutathione reductase levels suggest better antioxidant capacity, associated with less metabolic damage, and the absence of hypertension also clearly contributes to the classification as healthy.

Figure 12B provides additional context by illustrating the direction of each variable’s effect. Blue dots indicate low values, while red dots indicate high values.

For most healthy patients, low values for Years with DM2 and HbA1c are located to the left of the axes, indicating a healthy classification. In contrast, when these variables increase, the points shift to the right, reducing the probability of being classified as healthy. Similarly, low values for inflammatory markers (such as IL-18) and oxidative stress align with a healthy profile, whereas high levels shift the prediction toward more clinically severe states. From a clinical perspective, these results are consistent.

(2) SHAP diagnosis of T2DM patients

**Figure 13:**
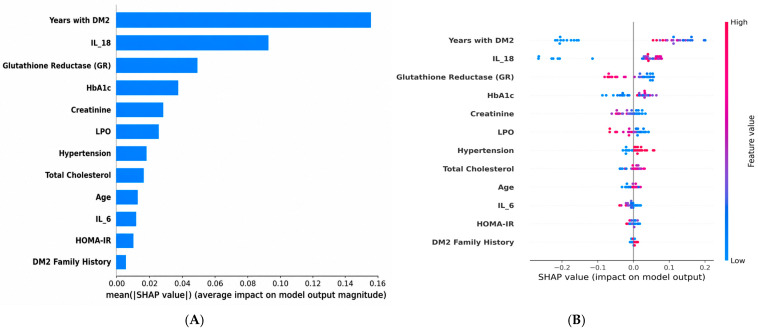
(**A**) Global feature importance for the class Diabetes (SHAP). (**B**) Feature impact on predicting Diabetes class (SHAP).

The SHAP analysis indicates that the model identifies T2DM patients primarily based on variables reflecting disease progression, systemic inflammation, and glycemic control. The most influential variable was “Years since T2DM diagnosis,” indicating that disease duration is the strongest determinant guiding the model’s classification within the diabetes category. Longer disease duration was associated with a higher probability of remaining in this class (See Figure 13A).

Complementarily, IL-18 and Glutathione Reductase (GR) highlight the central role of inflammation and oxidative stress. The presence of IL-18 values and alterations in GR increased the probability of diabetes classification, suggesting a persistent inflammatory metabolic state. Likewise, HbA1c contributes significantly, underscoring that sustained poor glycemic control remains a critical factor. Other variables (creatinine, LPO, hypertension, total cholesterol, and age) provided additional information but contributed relatively little. Taken together, the model captures a consistent pattern in which disease duration, inflammation, and chronic hyperglycemia collectively drive diabetes classification (See Figure 13B).

(3) SHAP diagnosis—patients with complications (micro/macro)

**Figure 14:**
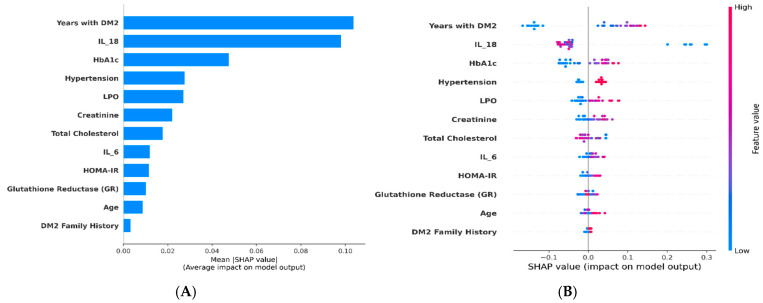
(**A**) Global feature importance class complications (SHAP). (**B**) Feature impact on predicting class complications (SHAP).

Among complicated patients, the model’s behavior changes, becoming more “aggressive” in clinical terms. Once again, “Years with T2DM” ranks first, reinforcing the idea that the risk of complications is cumulative. However, here, the roles of IL-18 and HbA1c become more relevant: higher values strongly shift the prediction toward the complications group, suggesting that chronic inflammation and poor glycemic control accelerate tissue damage. In addition, hypertension and markers of oxidative stress (such as LPO and creatinine) are positioned as determining factors (See Figure 14A,B). The presence of hypertension particularly increases macrovascular risk, while elevated creatinine levels are associated with renal impairment and progression to microvascular damage.

These results are clinically consistent: the model recognizes that complications arise when disease duration, sustained inflammation, oxidative stress, and metabolic dysregulation converge (see Table 11). In summary, SHAP analysis demonstrates that the model does not operate as a “black box” but instead produces results consistent with established pathophysiological patterns reported in the literature. Specifically, the factors that contribute to disease progression are the same ones that drive the development of complications.

## 5. Discussion

Machine learning is a valuable and innovative approach to healthcare, offering strong predictive capabilities for analyzing complex datasets. In this study, two populations with the same clinical condition (T2DM and cardiovascular complications) were combined to develop a predictive framework. To our knowledge, this dataset is among the few that integrate clinical, inflammatory, molecular, and oxidative stress markers in T2DM patients across different populations. Across both cohorts, we identified valuable predictive molecular markers associated with microvascular and macrovascular complications.

The proposed methodology demonstrates that a reduced but highly informative subset of variables can accurately stratify patients with T2DM and cardiovascular diseases. The use of correlation as a feature-selection method, combined with a two-stage modular model (overall classification followed by complication-type prediction), enhances model interpretability and clinical relevance. Further improvements in the model’s performance are expected with larger, more balanced, and more diverse datasets.

Unlike studies that primarily focus on algorithmic innovation, this work emphasizes integrating diverse biomarker domains and ensuring their clinical interpretability. The proposed hierarchical framework enables not only classification but also clinically relevant stratification of vascular complications, thereby supporting decision-making in personalized medicine.

Several studies have attempted to predict cardiovascular disease (CVD); however, all rely on clinical and biochemical features. According to the results of [37], a review article, ten studies focused on machine learning, reporting that neural network (NN) models achieved 76.6% precision, 88.06% sensitivity, and an area under the curve (AUC) of 0.91 in predicting cardiovascular disease (CVD) risk among patients with type 2 diabetes mellitus (T2DM).

It is important to emphasize that these studies did not incorporate lipid peroxidation markers. In contrast, our work includes oxidative stress markers, such as GPx, which are among the 10 clinically significant predictive variables. GPx, together with GRd, plays a critical biological role by inhibiting lipid peroxidation, thereby protecting cellular membranes and organelles from oxidative damage.

Additionally, Xu, C et al. [38] analyzed 4015 T2DM patients, including 999 with CVD, using the National Health and Nutrition Examination Survey (NHANES) dataset (1999 to 2018). Six ML models were trained, and XGBoost demonstrated the best performance (AUC = 0.75 on the training dataset and AUC = 0.72 in the test dataset), suggesting good generalization and making it more suitable for clinical applications. The Boruta feature selection algorithm was used to determine key clinical predictors, including estimated glomerular filtration rate (eGFR), age, total cholesterol (TC), low-density lipoprotein (LDL), creatinine, and blood urea nitrogen (BUN), which had the highest feature importance rankings consistent with our findings, total cholesterol also emerged as a highly predictive variable within the RF model and Pearson correlation performance.

On the other hand, Chen et al. [39] incorporated a distinct set of features for predicting the risk of coronary heart disease (CHD). The study enrolled patients with T2DM who underwent coronary angiography (CA) and were either diagnosed with CHD or had only T2DM. Five machine learning algorithms were trained, with the XGBoost model demonstrating optimal performance based on selected features: HbA1c, creatinine (Crea), aspartate aminotransferase (AST), lipoprotein a (Lp(a)), apolipoprotein Ai (Apo Ai), hypertension, smoking status, age, fibrinogen (FIB), high-density lipoprotein cholesterol (HDL-C), albumin (ALB), glucose (Glu), and total protein (TP). The XGBoost model achieved an area under the curve (AUC) of 0.814 (95% CI, 0.779–0.847), accuracy of 0.799 (95% CI, 0.771–0.827), precision of 0.841 (95% CI, 0.812–0.868), recall of 0.920 (95% CI, 0.898–0.941), and F1-score of 0.879 (95% CI, 0.859–0.897) in the testing set. While hypertension and HbA1c were standard features in the present studies, Chen et al. identified Lp(a) and Apo(Ai) as the most important features in their optimal model. These markers represent LDL- and HDL-specific particles, respectively, which are strongly associated with cardiovascular disease. Consistent with our results, hypertension and HbA1c were among the top 10 Gini importance features for the RF classifier in both micro and macro settings, and they showed a strong positive Pearson correlation.

Although Pearson correlation was used as an initial filtering step to reduce redundancy among variables, it primarily captures linear relationships and may not fully represent complex nonlinear interactions. In addition, no formal multicollinearity analysis (e.g., variance inflation factor) was conducted. While RF is relatively robust to multicollinearity in predictive performance and can model nonlinear dependencies, correlated predictors may still undermine the stability of feature-importance estimates and the interpretability of SHAP values. Therefore, future studies should incorporate formal assessments of collinearity and explore alternative feature selection methods that account for nonlinear relationships to improve model robustness and interpretability. This study evaluated the predictive value of multiple inflammatory cytokines and redox biomarkers for metabolic risk and the development of vascular complications in T2DM. Among all the markers studied, IL-18 emerged as the biomarker with the most excellent discriminatory power, both in classical analyses (ROC/logistic regression reported in the literature) and in the advanced machine learning models developed in this study, where IL-18 was the variable with the most significant overall importance and the initial division point in the tree representing the RF model.

Evidence from our previous work in the European population, Rusanova et al. [22], shows that, when analyzing diabetic patients with and without complications, the AUC of IL-18 (0.720) slightly exceeds that of IL-6 (0.708), indicating that both cytokines are relevant predictors and that IL-18 provides finer discrimination among patients with established vascular damage. This finding is consistent with the biological nature of IL-18, a cytokine typically activated by the NLRP3 inflammasome, which is highly sensitive to sterile inflammation, such as oxidative stress, and to chronic metabolic dysfunction characteristic of T2DM. Unlike IL-6, which is strongly modulated by adiposity, fat mass, and visceral adipose tissue, IL-18 reflects central mechanisms of inflammatory activation mediated by IL-1β and caspase-1, which are more closely linked to vascular deterioration, endothelial dysfunction, and chronic organ damage [40].

Mechanistic evidence summarized in the IL-18 review [41], supports this interpretation, indicating that IL-18 acts as a key downstream effector of the inflammasome, amplifies sterile inflammation, promotes endothelial activation and vascular stiffening, and, in some models, contributes to proapoptotic processes in metabolically sensitive tissues. Moreover, genetic and Mendelian randomization studies suggest that sustained elevation of IL-18 is not only associated with but may also causally contribute to cardiometabolic risk and atherosclerosis, through multiple comorbidities (obesity, fatty liver, chronic stress), and is, although predictive, less specific for the vascular deterioration characteristic of T2DM.

The DT analysis revealed a hierarchical organization of biomarkers, suggesting biologically plausible progression from systemic inflammation toward vascular dysfunction in T2DM. Disease duration appeared as an initial stratification factor, indicating that cumulative metabolic exposure plays a key role in shaping downstream phenotypic patterns. Within this context, IL-18 emerged as an early discriminative node, supporting its role as a marker of systemic inflammatory burden rather than a direct determinant of vascular deterioration.

Importantly, although IL-18 contributed to early stratification, final classification into diabetic or vascular phenotypes depended primarily on oxidative stress and endothelial-related markers, including glutathione peroxidase (GPx), superoxide dismutase (SOD), catalase, and nitric oxide (NO). This suggests that systemic inflammation alone may be insufficient to explain phenotypic progression, highlighting redox imbalance as a proximal mechanism.

Interestingly, IL-6 retained substantial predictive value, particularly when combined with microRNAs (miR-21, miR-126) and oxidative biomarkers, consistent with findings from our previous publication, in which several multivariate models, including IL-6, achieved AUC values between 0.79 and 0.81. However, its clinical specificity may be lower due to its strong association with nonspecific systemic inflammation, adiposity, and generalized proinflammatory states. Indeed, IL-6 is well known to be closely linked to being overweight and obesity. In the present study, all groups had BMI values above 25, with no significant anthropometric differences, suggesting that IL-6 levels may primarily reflect adiposity-related inflammation rather than risk of vascular complications. Consistently, in our second study involving a Spanish population, IL-6 did not demonstrate significant predictive value for the development of cardiovascular complications.

A key finding is that ML models, by exploring nonlinear and multidimensional relationships, provide complementary insights beyond classical statistics. The identification of IL-18 as a major decision node suggests that it is a highly discriminative variable across complex clinical categories and retains high discriminative power even in the presence of multiple clinical, metabolic, and molecular variables. This behavior aligns with its underlying pathophysiology and indicates that IL-18 may capture aspects of cardiometabolic risk not fully reflected by conventional biomarkers.

Furthermore, the integration of IL-18 with antioxidant system enzymes (such as GRd or G6PD) reinforces the concept that inflammasome-mediated inflammation is tightly linked to oxidative stress pathways [22], highlighting that the disruption of redox balance is a central axis of metabolic deterioration.

Based on the present findings and the evidence in the literature, IL-18 [42] should be included in predictive clinical models and risk stratification in T2DM, because:

It demonstrated a higher AUC than IL-6 for distinguishing between complicated and uncomplicated diabetes.

It reflects central causal mechanistic pathways involving inflammasome activation and sterile inflammation.

It appears less dependent on adiposity-related variability, potentially improving stability across diverse populations.

It improves ML models and ranks as the most crucial variable in nonlinear classifiers.

It presents a dual clinical pattern (elevated vs. depressed) that may indicate distinct states of inflammatory dysfunction, thereby increasing its sensitivity to detect risk subphenotypes.

The integration of IL-18, miR-126, miR-21, IL-10, and nitric oxide into both traditional clinical models (years with T2DM, HbA1c, urea, total cholesterol, and hypertension) and AI-based predictive frameworks represents a promising step forward in personalized medicine [43]. This approach enables more precise detection of patients at risk of vascular complications, potentially even before conventional clinical or biochemical markers become evident.

These findings are consistent with recent studies highlighting the value of integrating clinical and novel protein biomarkers to improve predictive performance for T2DM-related complications described by Francis et al. [44]. However, unlike approaches that rely solely on clinical variables, our model incorporates multimodal data, potentially enhancing interpretability and clinical relevance.

Despite these promising findings, several limitations should be acknowledged. First, integrating cohorts from different geographical regions may introduce cohort-specific biases related to population characteristics, laboratory conditions, or data acquisition processes. Although standardized clinical criteria were applied, no explicit batch correction or cohort adjustment was performed, which may have affected model consistency. Future studies should incorporate cohort-stratified validation or leave-one-cohort-out analyses to better assess model generalizability across populations.

Second, although the dataset used in this study is relatively small (*n* = 169), it represents a unique and clinically grounded cohort that integrates anthropometric, biochemical, inflammatory, molecular, and oxidative stress biomarkers obtained directly from patients in two populations (Mexico and Spain). To the best of our knowledge, no comparable dataset with this level of multimodal integration is currently available in the clinical context.

While ensemble models such as RF may be prone to overfitting when trained on small datasets, several strategies were implemented to mitigate this risk. Specifically, stratified cross-validation and bootstrap resampling were employed, both of which showed consistent performance across folds and low variability in the estimated metrics, supporting the model’s stability.

Another limitation concerns the handling of missing data. Missing values were addressed using a complete-case analysis approach, resulting in the exclusion of 11 participants from the original cohort. Although this strategy avoids introducing uncertainty associated with imputation methods, no formal assessment of the missing-data mechanism (e.g., Missing Completely At Random, MCAR) was performed. Consequently, the potential impact of missing-data patterns and selection bias cannot be entirely ruled out. Future studies should evaluate the underlying mechanism of missingness and compare complete-case analysis with alternative approaches, such as multiple imputation, to assess the robustness of the results.

Additionally, the decision tree structure (Figure 10) reveals clinically interpretable patterns based on relevant biomarkers (e.g., years with T2DM, IL-18, SOD), suggesting that the model captures meaningful physiological relationships rather than noise. Although some terminal nodes contain a limited number of samples, this is a common characteristic in clinical datasets and reflects the inherent heterogeneity of patient populations rather than overfitting.

Therefore, the reported results should be interpreted as exploratory, and further validation in larger, independent, and more diverse cohorts is required to confirm the robustness and generalizability of the proposed approach.

Additionally, the present study does not include confidence intervals for performance metrics, formal statistical comparisons between models, or precision–recall curves, which are particularly relevant in imbalanced clinical datasets. These analyses were not included due to the limited sample size, which may affect their stability and interpretability. Future work should incorporate these statistical evaluations into larger cohorts to provide a more comprehensive assessment of model performance. Future studies should explore additional class-balancing strategies, including synthetic oversampling techniques such as SMOTE and ADASYN, as well as other resampling methods, to improve the identification of underrepresented microvascular complications.

## 6. Conclusions

An RF approach, enhanced by correlation-driven feature selection, accurately classifies patients into healthy, diabetic, or diabetic with complications groups. Incorporating a secondary predictor for complication type further improves clinical insights and enables more detailed patient stratification. This strategy supports decision tools that provide personalized T2DM care.

Clinical conclusions from the model indicate that the duration of a patient’s diabetes is the primary trigger. Subsequent changes in inflammatory and oxidative stress biomarkers mark the onset of complications. Rather than a single cause, multiple interacting factors contribute to disease progression. This pattern aligns with the recognized clinical course of T2DM and supports the use of a layered analytical approach. The analysis demonstrates that RF models not only classify but also uncover significant biological relationships and offer robust quantitative support for clinical decision-making.

The proposed approach demonstrates promising performance for stratifying patients with T2DM using multimodal biomarkers. However, these findings should be interpreted as exploratory, given the absence of external validation and benchmark comparisons. Future work should include evaluation against baseline models and validation on independent datasets to further assess the robustness and generalizability of the proposed framework.

The model was evaluated using stratified cross-validation, which allows estimating its performance under internal data partitions. However, this approach does not fully address potential distribution shifts across different clinical populations. To strengthen this analysis, a sensitivity study was conducted by applying ±10% perturbations to numerical features, showing that the model maintains stable performance (accuracy = 0.9431), suggesting robustness to moderate variations in the data.

Additionally, a distribution shift analysis was performed using HbA1c by dividing the dataset into subgroups based on its median value. The model was trained on one subgroup (low HbA1c) and evaluated on the other (high HbA1c), achieving an accuracy of 0.7561. In the reverse scenario, accuracy decreased to 0.3043. These results indicate that although the model performs well on internal validation, its generalization capability may be affected by shifts in the distribution of key clinical variables.

Consequently, variables such as diabetes duration, HbA1c, and IL-18 may exhibit different behaviors across independent cohorts, potentially impacting model performance. Therefore, external validation using independent datasets is recommended, along with the exploration of domain adaptation techniques in future work.

## Figures and Tables

**Table 1:** Confusion matrix and metrics.

		Predicted Class		
		Positive	Negative	
**Actual Class**	**Positive**	(TP)	(FP)	**Recall** *TP*/*TP* + *FN*
	**Negative**	(FN)	(TN)	**Specificity** *TN*/*TN* + *FP*
		**Precision** *TP*/*TP* + *FP*	**Negative Predictive Value** *TN*/*TN* + *FN*	**Accuracy** $\frac{TP+TN}{\left(TP+TN+FP+FN\right)}$

Where True Positive (TP) refers to a positive case correctly classified as positive; False Positive (FP) denotes a negative case incorrectly classified as positive; False Negative (FN) represents a positive case incorrectly classified as negative; and True Negative (TN) corresponds to a negative case correctly classified as negative. The following performance metrics were calculated from these categories.

**Table 2:** Absolute values of the correlation between variables and class.

Variable	Absolute Correlation with Class
1. Years with T2DM	0.629343
2. Hypertension	0.599595
3. HbA1c	0.577679
4. Glutathione Reductase (GR)	0.492493
5. miR-21	0.422414
6. DM2 Family History	0.418601
7. Age	0.372742
8. Total Cholesterol	0.354978
9. HOMA-IR	0.348969
10. AOPP	0.342859
11. Physical Activity	0.341776
12. GSH	0.335651
13. Glutathione Peroxidase	0.328038
14. Catalase	0.306513
15. Urea	0.298740
16. G6PDH	0.289459
17. GSSG_GSH	0.277226
18. BMI	0.273957
19. SOD	0.271258
20. miR-126	0.261618
21. IL_6	0.240748
22. LPO	0.222951
23. IL_10	0.210937
24. Creatinine	0.180343
25. Triglycerides	0.176731
26. IL_18	0.168934
27. NO	0.126673
28. Gender	0.119867
29. GSSG	0.093751
30. TNF_alfa	0.029303
31. Smoking	0.011344

**Table 3:** Top correlations matrix.

Variable	Absolute Correlation
Years with T2DM	0.474212
Hypertension	0.454364
Total Cholesterol	0.421252
miR-21	0.382166
Glutathione Peroxidase	0.354085
HbA1c	0.342536
IL_18	0.302105
Age	0.284667
IL_10	0.265438

Table 3 summarizes the strongest correlations observed among clinical, biochemical, molecular, inflammatory, and oxidative-stress biomarkers associated with T2DM complications.

**Table 4:** Performance comparison and statistical significance of the evaluated classifiers.

Model	Macro F1 Score	SD	*p*-Value (vs. RF)	Holm-Adjusted *p*-Value	Significant
RF	0.7994	0.0589	—	—	Reference
DT	0.7916	0.0618	3.23 × 10^−3^	3.23 × 10^−3^	Yes
NB	0.7878	0.0643	5.43 × 10^−4^	1.09 × 10^−3^	Yes
SVM	0.7646	0.0624	5.01 × 10^−25^	1.50 × 10^−24^	Yes
MLP	0.7303	0.0631	3.43 × 10^−62^	1.37 × 10^−61^	Yes
LR	0.7117	0.0686	1.88 × 10^−65^	9.39 × 10^−65^	Yes
LDA	0.7084	0.0704	3.78 × 10^−66^	2.27 × 10^−65^	Yes
QDA	0.6998	0.0839	1.34 × 10^−71^	9.37 × 10^−71^	Yes

Performance comparison of the evaluated classifiers using repeated stratified cross-validation. Statistical significance was assessed using the Wilcoxon signed-rank test with Holm correction, taking RF as the reference model. All adjusted *p*-values were below 0.05, indicating significant differences between RF and the remaining classifiers.

**Table 5:** Comparative model performance (validation).

Model	Accuracy	Precision	Recall	F1 Score
RF	0.943137	0.943175	0.942222	0.941989
DT	0.909150	0.916984	0.906667	0.906596
GB	0.898039	0.915238	0.895556	0.892954
SVM	0.873856	0.884841	0.871111	0.871138
MLP	0.850327	0.865238	0.844444	0.846177
NB	0.839869	0.856508	0.833333	0.831758
LDA	0.816340	0.836667	0.806667	0.804634
LR	0.805882	0.808413	0.797778	0.795130
QDA	0.507843	0.492896	0.497778	0.466328

The table shows the comparative performance of nine ML models evaluated using stratified cross-validation for the diagnostic classification of patients (H, D, and C). The metrics considered include accuracy, precision, recall, average F1 Score, and the standard deviation of the F1 score across folds, allowing assessment of both classification performance and algorithm stability.

**Table 6:** Variable importance derived from the Random Forest model (Stage 1 classification) dataset.

Variable	Importance	Interpretation
IL-18	0.112853	Pro-inflammatory biomarker associated with vascular damage.
miR-126	0.050626	microRNA linked to endothelial dysfunction and vascular complications.
Years with T2DM	0.049545	Longer disease duration increases complications risk.
HbA1c	0.038321	Marker of long-term glycemic control.
IL-10	0.035372	An anti-inflammatory cytokine involved in immune regulation.
Urea	0.031109	A marker of renal dysfunction.
miR-21	0.028083	microRNA associated with inflammation and tissue remodeling.
NO	0.025347	An indicator of endothelial function and oxidative stress.
Total Cholesterol	0.021313	Associated with cardiovascular risk.
Hypertension	0.020785	Risk factor for vascular complications.
G6PDH	0.018854	Enzyme involved in oxidative stress regulation.

This table provides a simplified view of the RF model’s internal decision-making process for identifying complex patients. It shows that clinical and inflammatory variables such as IL-18, miR-126, years with T2DM, and HbA1c play a central role in early classification decisions. Although the complete model comprises multiple trees, this visualization allows us to understand how automated decisions are made from real clinical data, thereby facilitating both model validation and medical interpretability.

**Table 7:** Classification report (Stratified 5-fold CV, all individuals).

Class	Precision	Recall	F1-Score	Support
Healthy	1.00	1.00	1.00	12
Diabetic	0.69	0.82	0.75	11
Complicated	0.78	0.64	0.70	11
Accuracy			0.82	34
Macro avg	0.82	0.82	0.82	34
Weighted avg	0.83	0.82	0.82	34

While cross-validation yielded an average accuracy of 0.92, evaluation on the held-out test set resulted in an overall accuracy of 0.82, reflecting more realistic performance under unseen-data conditions.

**Table 8:** Distribution of complications in the Stage 2 dataset.

Complication Type	Number of Cases
Microvascular	26
Macrovascular	30
Total	56

**Table 9:** Variable importance derived from the Random Forest model (Stage 2 classification) dataset.

Variable	Importance	Interpretation
LPO	0.120425	A metabolic process that causes oxidative deterioration of lipids by ROS.
IL-18	0.098996	Pro-inflammatory biomarker associated with vascular damage.
Creatinine	0.090723	A breakdown product of creatine phosphate from muscle and protein metabolism.
GSH	0.071917	Defends cells against free radicals and oxidative stress.
GSSH_GSH	0.068231	Ratio is a primary clinical biomarker used to measure systemic oxidative stress.
IL_6	0.065330	It acts as an important driver of the acute inflammatory response.
GR	0.062410	Responsible for maintaining the supply of reduced glutathione.
HbA1c	0.059676	Marker of long-term glycemic control.
Urea	0.057670	A marker of renal dysfunction.
IL_10	0.051602	Associated with cardiovascular risk.
Years with DM2	0.050800	Risk factor for vascular complications.

ROS: Reactive Oxygen Species.

**Table 10:** Performance metrics for Stage 2 classification.

Class	Precision	Recall	F1-Score	Support
Microvascular	1.00	0.14	0.25	7
Macrovascular	0.79	1.00	0.88	23
Accuracy			0.80	
Macro Avg	0.90	0.57	0.57	30
Weighted Avg	0.84	0.80	0.74	30

**Table 11:** Global SHAP Importance Across Classes (H/D/C).

Importance SHAP Average of Absolute Value	Healthy	Diabetic	Complicated
Years with T2DM	0.2515	0.1477	0.1037
HbA1c	0.0756	0.0338	0.0476
Glutathione Reductase (GR)	0.0431	0.0436	0.0102
Hypertension	0.0427	0.0164	0.0277
IL_18	0.0134	0.0851	0.0981
Age	0.0133	0.0105	0.0087
LPO	0.0067	0.0217	0.0271
HOMA-IR	0.0061	0.0080	0.0115
DM2 Family History	0.0048	0.0041	0.0033
Total Cholesterol	0.0040	0.0150	0.0177
IL_6	0.0030	0.0095	0.0118
Creatinine	0.0023	0.0233	0.0221

The specific analysis of the Complications variable showed that IL-18, total cholesterol, hypertension, IL-6, and other metabolic and oxidative stress markers have the strongest associations with the presence and type of complications in patients with T2DM.

## Data Availability

Data supporting the reported results are available at: https://github.com/gggvamp/T2DM (accessed on 20 June 2026) (Supplementary Materials).

## Supplementary Materials

The following supporting information can be downloaded at: https://github.com/gggvamp/T2DM (accessed on 20 June 2026).
